# Slovenian Consumer Food Safety Study: Knowledge, Attitudes, and Practices from Shopping to Preparation Based on Questionnaire Analysis

**DOI:** 10.3390/foods14244215

**Published:** 2025-12-08

**Authors:** Maja Bensa, Mojca Jevšnik Podlesnik, Lato Pezo, Irena Vovk

**Affiliations:** 1 Research Institute of Faculty of Health Sciences, Faculty of Health Sciences, University of Ljubljana, 1000 Ljubljana, Slovenia; 2 Department of Sanitary Engineering, Faculty of Health Sciences, University of Ljubljana, 1000 Ljubljana, Slovenia; 3 Institute of General and Physical Chemistry, University of Belgrade, 11000 Belgrade, Serbia; latopezo@yahoo.co.uk; 4 Laboratory for Food Chemistry, National Institute of Chemistry, 1000 Ljubljana, Slovenia; irena.vovk@ki.si

**Keywords:** food safety, food safety culture, food safety knowledge, food safety attitudes, food safety practices, food labeling, use by date, refrigerator storage temperature, questionnaire, structural equation modeling

## Abstract

Foodborne diseases remain a persistent public health problem. Most foodborne outbreaks in Europe occur in consumers’ homes, highlighting that improvements in consumer food safety are needed and that consumers have an important role in maintaining food safety. A better understanding of consumer food safety knowledge, attitudes, and food handling practices is required to prepare effective interventions. The aim of this study was to evaluate consumer food safety knowledge, attitudes, and food handling practices in Slovenia as well as the interrelationships between knowledge, attitudes, and practices. Adult consumers in Slovenia (*n* = 1621) participated in a validated online questionnaire. The questionnaire included demographic characteristics and explored the aspects of knowledge, attitudes, and practices on the following topics: food related habits, food shopping and transportation, food refrigeration, food labeling, and food preparation. Data were analyzed using descriptive statistics and structural equation modeling (SEM). Overall, participants demonstrated good levels of knowledge, attitudes, and practice. However, some of the areas that require improvement include use of cooling bags, measuring and knowing the correct refrigerator temperature and not washing raw meat and poultry. SEM analysis revealed that knowledge affects attitudes and, in most cases, both knowledge and attitudes affect practices. Enhanced communication strategies targeting consumers are needed to reduce the risk of foodborne illnesses. Educational campaigns need to focus on all three aspects: knowledge, attitudes, and practices. Building a culture of consumer food safety is essential because consumer food safety truly is everyone’s business.

## 1. Introduction

Food safety is a global public health problem. Some simple statistics point out the extent of the problem. It is estimated globally that every year, one in ten people will become sick due to consumption of contaminated food [1]. It is also estimated globally that the consumption of contaminated food results annually in 600 million people becoming ill, with over 400,000 deaths and not to mention the economic costs of over 100 billion USD [1]. The consequences of foodborne diseases clearly negatively impact the society, the health sector, and the economy. Although there are many reasons for foodborne outbreaks, there are numerous scenarios by which consumers, as the last stakeholder in the food safety chain, can make a big difference. Food safety also remains a significant public health concern in Europe, where the European Food Safety Authority (EFSA) has found that the primary place of exposure to foodborne outbreaks is in domestic premises (2016–2023 and in 2022, with an equal number of outbreaks in homes and restaurants) [2], demonstrating that consumers have an important role in ensuring food safety. In Slovenia, the latest publicly available data from 2023 reported 6387 cases of intestinal infectious diseases, including noroviruses (over 1600 cases), campylobacteriosis (over 1000 cases), *Clostridium difficile* infections, *Escherichia coli* infections, rotaviruses, and salmonellosis [3]. Unfortunately, this epidemiological data does not include information about the number of outbreaks that occurred in consumers households. Most of the food safety chain is governed by regulations and inspections from production through retail. However, since consumers are basically “home alone”, food safety relies on the consumer’s knowledge, attitudes, and practices of food safety (KAP). Enhanced understanding of consumer food safety KAP is essential for improving consumer food safety and public health. So, if the bad news is that there are many foodborne outbreaks causing health problems and in Europe most foodborne outbreaks occur in consumers’ homes, the good news is that there are simple solutions which consumers can adopt to improve their food safety and help prevent foodborne illnesses.

Studies of consumer food safety have been conducted on several continents, focusing on multiple issues in consumers’ food handling by investigating either one (K, A or P) or a combination of the KAP aspects of food safety. All three KAP aspects were investigated in some of the studies in Ireland [4], Sweden [5], Romania [6], Jordan [7], and Malaysia [8]. Other studies examined knowledge, risk perceptions (which can be considered a component of attitude), and practices in the USA [9,10], United Arab Emirates [11], and Italy [12]. Some of the studies focused only on specific population groups (instead of consumers in general), such as the elderly [13], women [7,11,14], university students [5], high school students together with their parents and teachers [12], as well as elementary school students [15]. In studies of consumer food safety, the information was most frequently collected using questionnaires. Other methods included (1) interviews [16,17], (2) focus groups [18], and (3) mixed methods (observations, kitchens mapping, photos, and interviews [19]; questionnaire, observations, and kitchen inspections with obtaining surface samples [20]; interviews and observations [21]). To maintain food safety throughout consumer food handling, it is important to take care of good hygiene, prevent cross-contamination, and keep food at appropriate temperatures. The research questions focused on the following food handling topics: **(1) food shopping** [9,11] **and transport** [11], **(2) cross-contamination during refrigerator storage** [4,5,8,10,11], **(3) refrigerator temperature** [4,5,9,10,11], **(4) reading and following food product information on labels (e.g., expiration dates and instructions for storage and preparation** [8,10,11]**)**, **(5) cross-contamination during cooking and food preparation (cross-contamination** [4,8,9,11,12], **cooking when sick or with a hand wound** [8,15,22,23,24,25,26,27,28,29,30,31]**)**, **(6) washing fruits and vegetables** [6,11], **and (7) washing meat and poultry before cooking** [4,6,8,9]. Some studies covered these topics from perspectives of a single pathogen such as *Salmonella* [4] or specific foods such as poultry [32] or fresh produce [10]. In addition to assessing the levels of KAP, the **relationships among KAP** were also investigated in Malaysia [8,33], Lao People’s Democratic Republic [34], Romania [6], and Sweden [5].

Differences in research approaches (methodology, questions, population, sample size, countries) and conclusions of the published studies clearly reveal knowledge gaps that need to be addressed. These gaps include a better understanding of (1) the connections and relationships among aspects of KAP, (2) the levels of KAP, and (3) consumer food safety in localities where it has not been investigated comprehensively. Furthermore, in Slovenia this is evident, as the previous research on consumer food safety did not include a systematic overview of knowledge about food safety, attitudes towards food safety, and practices or behavior of consumers on the entire scale of consumers food handling (e.g., from purchase, storage, preparation of food, to storage of food leftovers, etc.). So far, research in this field in Slovenia has been limited to (1) mostly only knowledge about food safety and food handling practice, (2) mostly a small number (less than 600) of research participants, (3) specific population groups, and (4) only certain risk factors for food safety, and has not yet applied structural equation modeling to investigate the effects between KAP. The aim of this study is to address these gaps by conducting a mixed-methods investigation of consumer food safety among adults in Slovenia. This paper reports the first segment of the questionnaire results: (1) levels of KAP across a broad range of food handling activities from shopping through food preparation, and (2) relationships among the aspects of KAP analyzed using structural equation modeling (SEM). These findings provide new insights into consumer behavior in Slovenia and can serve as an important foundation for creating targeted strategies to improve consumer food safety in Slovenia and beyond.

## 2. Materials and Methods

### 2.1. The Consumer Food Safety Study and Questionnaire Design

This Consumer Food Safety Study (CFSS) was approved by the University of Ljubljana Biotechnical Faculty Ethical Committee (“Komisija za etično presojo raziskav s področja prehrane (KEP)-KEP-3-12/2023”). The aim of the CFSS was to evaluate two aspects: (1) the levels of food safety related knowledge, attitudes, and food handling practices, and (2) the effects of knowledge on attitudes and practices as well as the effects of attitudes on practice.

The investigation was conducted with a validated questionnaire including the research questions created based on the **Matrix of Consumer Food Safety (MCFS)**. MCFS explored knowledge, attitudes, and practices (KAP) through a wide range of consumer food handling activities ranging from shopping through food preparation. MCFS is the result of a systematic approach to questionnaire design aiming to provide relevant questions related to KAP for the food safety topics.

Development of the MCFS was performed in steps. First, important factors for food safety ((1) appropriate hygiene, (2) prevention of cross-contamination, and (3) keeping foods at appropriate temperatures) were considered together with consumer food handling activities. This led to the identification of critical points in consumer food safety throughout food handling. Then, based on these critical points the questions investigating KAP were developed. Together, the topics arising from critical points and the questions covering KAP resulted in the MCFS. This design simplified the investigation of effects of knowledge on attitudes and practices, as well as the effects of attitudes on practice. Possible MCFS applications include the following: (1) development of questionnaires and interviews, (2) development of more standardized questionnaires to enable enhanced comparisons across time and geographic locations, and (3) preparation of educational campaigns for consumers. A part of the MCFS covering the questions presented in this paper is provided in Appendix A (Table A1).

The questionnaire was written in Slovene, but participation was not limited to Slovenian citizens. The questionnaire included 60 compulsory questions of different types including Likert scales (1–5), multiple choice, and open-ended responses. Specifically, 1 corresponded to “not important at all” or “strongly disagree” or “never” and 5 corresponded to “very important” or “strongly agree” or “always”, depending on the context of the item. Intermediate points (2–4) represented gradations between these extremes, allowing participants to express varying levels of agreement or frequency. This scale was applied consistently across all items in the questionnaire. There were seven topical sections in the questionnaire: (1) demographic, (2) food safety (information, education and experience), (3) food shopping, transportation and refrigeration, (4) washing hands and cleaning the kitchen, (5) food labeling and food preparation, (6) thawing, heat treatment of food and leftovers, and (7) pets. The questionnaire included four demographic questions: (1) gender, (2) age, (3) education (last completed level of schooling), and (4) household members who are more vulnerable to foodborne disease (younger children (less than 6 years), persons older than 65 years, persons with chronic diseases, and pregnant/breast-feeding mothers). The scope of this paper includes the questionnaire topical sections 3 and 5, presenting the following topics: food related habits, food shopping and transportation, food refrigeration, food labeling, and food preparation.

### 2.2. Pilot Study

The questionnaire was pilot-tested with 20 participants, of which 5 were subject matter experts and 15 were consumers belonging to three age groups (young, middle, and older). Minor modifications were made to the questions based on the feedback received in the pilot study. The reliability analysis of topical section 3 of the pilot questionnaire “Food shopping, transportation and the refrigerator” (22 items: K13–K17, A2–A5, P1–P13) yielded a Cronbach’s alpha of 0.820, indicating good internal consistency. Similarly, topical section 5 “Food labeling and food preparation” (22 items: K22–K28, A11–A14, P68–P78) showed good internal consistency, with a Cronbach’s alpha of 0.811.

### 2.3. Questionnaire Administration

The target audience for the questionnaire were consumers in Slovenia. Participants were required to be older than 18 years and represented different ages, genders, and educational backgrounds. Participation was not limited to Slovenian citizens. The questionnaire enabled collection of information from a large sample. Snowball sampling was used to invite participants to take part in the questionnaire and share the information about the study with other potential participants. Emails, fliers, and posters about the questionnaire were sent to **larger public institutions** (such as libraries, universities, research institutes, secondary schools) and **state institutions** (such as ministries) in Slovenia. The study was promoted on social media such as Facebook, LinkedIn, Instagram, and X/Twitter. The questionnaire was available from October 2023 to May 2024 on an online survey service called 1KA, developed by the University of Ljubljana Faculty of Social Sciences and available on https://www.1ka.si [35]. In order to access the questionnaire’s first question, the participants were required to read and tick a box that they gave their “Informed consent to participate in the study”.

### 2.4. Statistical Analysis

The statistical software IBM SPSS (Version 26) was used to analyze the questionnaire results. Descriptive statistics were used to evaluate the levels of KAP among participants, and the results were graphically presented with Microsoft Excel (Microsoft 365 MSO). The structural equation modeling (SEM) was used to test the KAP models using the IBM Amos 21 statistical program. The structural modeling method was used to test the hypothetical KAP model. This SEM method is a further development of multiple regression analysis, which can provide more meaningful and valid interpretations of results than alternative methods. In contrast to multiple regression, SEM allows the metric properties of the individual variables and their relationships to be analyzed simultaneously, which makes it much easier to interpret the results. In the SEM analysis, the latent constructs were defined based on thematic sections of the questionnaire, and the observed variables corresponded to individual questionnaire items. Model fit was evaluated using standard indices, including CFI (Comparative fit index), GFI (Goodness of fit), NFI (Normed fit index), and RMSEA (Root mean square error of approximation). Missing data was handled by listwise deletion to ensure complete data for all included cases.

## 3. Results and Discussion

### 3.1. Demographic Characteristics of Participants

**Figure 1:**
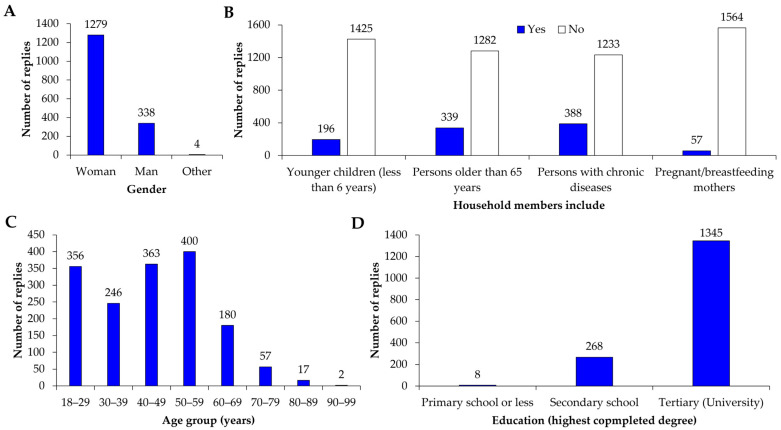
Demographics characteristics of study participants: gender (**A**), household members (**B**), age groups (**C**) and education (**D**).

The questionnaire was completed by 1621 participants. Among them were 78.9% (1279) women, 20.9% (338) men, and 0.2% (4) people identifying as other (Figure 1A). Some of the CFSS participants had household members who are more vulnerable to foodborne disease: 12.1% (196) had younger children (less than 6 years), 20.9% (339) had persons older than 65 years, 23.9% (388) had persons with chronic diseases, and 3.5% (57) had pregnant/breast-feeding mothers (Figure 1B). Participants ranged in age from 18 to 90, with the following age group representation: 21.96% in their 20s (ages 18–29), 15.18% in their 30s, 22.39% in their 40s, 24.68% in the 50s, 11.10% in their 60s, and 4.69% in their 70s to 90s (Figure 1C). The vast majority of the participants 82.97% (1345) achieved tertiary education (university or higher education), 16.53% (268) finished secondary school, and 0.49% (8) finished primary school or less (Figure 1D).

### 3.2. Levels of Knowledge (K), Attitudes (A), and Practices (P)—KAP

#### 3.2.1. Habits

Overall, the questions on habits (Table 1) showed that the vast majority of participants eat home-grown and bought food (66.6%), which is mostly cooked at home (72.9%) using basic ingredients (80.9%).

#### 3.2.2. Shopping and Transport

There are several consumer food safety aspects that come into play during food shopping and transport of food to consumers’ homes: (1) separating food items during shopping and transport (e.g., perishable foods together, dry foods together, etc.), (2) practices of buying unpackaged fruits and vegetables, (3) overall shopping order (where frozen and refrigerated foods should be last), (4) use of cooler bags/insulated bags to transport perishable foods home, and (5) time needed from checkout to transport perishable (refrigerated and/or frozen) foods to the refrigerator and/or freezer at home.

##### Food Separation During Shopping and Transport

While most of the CFSS participants strongly agreed or agreed with **separating foods** during shopping or transport, they showed stronger agreement with the separation of meat/poultry/fish from other foods than the separation of wet foods from dry foods (Table 2). The results of both questions demonstrate that the participants had a good level of knowledge about separating foods during shopping and transport. Also most of the university students from the USA (83%) knew that it is recommended to separate raw meat when shopping [9]. Over 60% of CFSS participants reported to always or almost always separate wet foods from other foods and separate raw meat/poultry/fish from other foods (Table 3). Similar findings were reported for consumers in Türkiye (66% separated fish from other foods) [36] and Ghana (over 66% always separated chicken from other vegetables and 52% from other groceries) [17]. In the U.S. Virgin Islands, 46% of consumers always separate raw beef from other foods [37]. In New Zealand, only 25% of consumers separate poultry in the trolley, shopping bag, and refrigerator [38].

##### Buying Unpackaged Fruits and Vegetables

Most CFSS participants (over 32%) use plastic bags when **buying unpackaged fruits and vegetables** in the store, followed by disposable gloves and plastic bags (almost 30%) (Table 1). Over 10% of participants use reusable bags, and almost 8% use disposable gloves and reusable bags (Table 1).

##### Shopping Order

CFSS participants are more concerned about the quick **transport** of frozen and perishable foods to their home (66.6% very important and 31.2% important) than about **adding these foods to the shopping basket last** (28.1% very important and 37.8% important) (Table 4). Attitudes about the shopping order of frozen and perishable foods could be improved, as 20.0% consider this to be neither important nor unimportant. Consumers from studies in other countries reported different opinions on the best time to buy frozen food. At the end of shopping time was the reply of 88% of women in Jordan [7] and 60% of consumers in Bangladesh [22]. In Poland, 26% of consumers replied correctly: first non-food products, followed by non-perishable food products and perishable foods requiring low temperatures [39]. Only 5% of consumers in New Zealand would buy raw poultry after shopping all other nonperishable food products [38].

Regarding **shopping order**, around 60% of CFSS participants always or almost always add frozen and perishable foods to their shopping basket last (Table 3). This shows an improvement compared to a previous Slovenian study where only 10% of Slovenian consumers purchased raw meat/tofu at the end of shopping [40]. Diverse shopping orders were reported in other countries. Perishable foods were purchased at the end of shopping by consumers in Saudi Arabia (16% of women—frozen products) [16], Poland (19%—fresh meat/fish/cured meats and 38%—frozen foods) [39], Egypt (28%—frozen ice cream) [24], U.S. Virgin Islands (37%—raw beef) [37], Ireland (50%—raw meat) [4], Lebanon (51%—raw meat [31] and 69%—chicken [25]), Türkiye (53%—fish) [36], Serbia (54–60% of university students—refrigerated meat) [23], and the United Arab Emirates (63% of women—frozen food [41] and 64% of women—refrigerated and cold foods [11]). In Poland, 49% of consumers let the product arrangement (store layout) dictate their shopping order [39]. In Saudi Arabia, 70% of women do not care when they purchase frozen products [16]. In the U.S. Virgin Islands, 55% of the consumers shop for raw beef straight away when they enter the store [37].

##### Use of Cooler Bags/Insulated Bags

Only around 36% of CFSS participants always or almost always use **cooler bags/insulated bags** to transport perishable foods (Table 3). Low usage of insulated bags among consumers (15% [40], 23% [42], and 40% (when buying poultry) [32]) was also reported in previous studies in Slovenia. In other countries, cooler bags/isolated bags were used by consumers in the U.S. Virgin Islands (28%—always) [37], Saudi Arabia (18% of women—always) [16], Poland (14%—always/usually for frozen foods) [39], and Ghana (12%—to transport fresh chicken) [17]. In Türkiye, more than one half of consumers paid attention to chill the fish during shopping [36].

##### Transport Time

The CFSS participants were quite quick to bring frozen or perishable foods from the store to their homes, as over 82% stated that it takes them up to 30 min to do this and over 13% stated that it takes them from 30 min to 1 h (Table 1). In other studies, the shortest **time for transporting** perishable foods was reported by consumers in Poland (40% needed 10–20 min) [39] and New Zealand (88% needed 0–40 min to place raw poultry in the refrigerator after shopping) [38]. Less than 1 h was needed by consumers in the United Arab Emirates (93% of women) [11] and the U.S. Virgin Islands (83%) [37]. Consumers usually needed less than 2 h to arrive home from the store in the United Arab Emirates (97% of women) [41], Türkiye (66%) [36], and Saudi Arabia (62% of women) [16].

#### 3.2.3. Refrigeration

##### Food Placement in the Refrigerator

To avoid cross-contamination in the refrigerator, the basic tips for proper food placement are related to the placement of fresh meat, poultry, and fish, which should be put on the lowest shelf in the refrigerator or separate from cooked food and food consumed raw. The CFSS participants had good knowledge regarding storing fresh meat, poultry, and fish on the lowest shelf in the refrigerator or separating them from cooked food and food consumed raw, as over 80% either strongly agreed or agreed with the importance of this separation (Table 2). Knowledge that **fresh meat, poultry, and fish should be stored on the lowest shelf** was reported for consumers in Ghana (11%—chicken in a sealed container) [17], Egypt (14%—raw meat) [24], Poland (29%—fresh ground meat) [39], Bangladesh (50%) [43], and Ireland (75%—fresh chicken in an airtight container) [4]. In Lebanon, the UK, and the USA, 90% of student dietitians knew that raw meat should not be stored above ready-to-eat foods [44]. Knowledge of **separating the fresh meat, poultry, and fish from cooked food and food consumed raw** was reported for consumers in the USA (10%—fresh cut fruits or bagged salad stored above raw meat and poultry) [10], Brazil (26%—raw meat and ready-to-eat foods [45]), and Poland (48%—raw meat and cured meat) [39], and 88% vegetables separate from animal products [46]).

The participants’ practices of placing foods in the refrigerator were quite good, as over 70% of CFSS participants separate foods that are consumed raw from foods that need to be heat-treated (35.3% always and 35.3% almost always) (Table 3). **Separate refrigerator storage of foods consumed raw from foods that need to be heat-treated** was reported for consumers in Bosnia and Herzegovina (62%) [27], the USA (70% of parents of 2–3 years old children) [47], India (87%: 61% always and 26% most of the time) [48], and Türkiye (38% never and 23% rarely placed fish on the same shelf as other foods) [36]. **Placing fresh meat, poultry, and fish on the lowest shelf** in the refrigerator was reported for consumers in Lebanon (24%—raw meat on the appropriate shelf) [31] and Italy (28% of students, parents and teachers—fresh meat on the bottom shelf) [12].

Furthermore, although the practices of storing fresh meat, poultry, and fish on the lowest refrigerator shelf or separate from cooked food and food eaten raw were slightly less good, the results still showed that over 66% of CFSS participants most of the time took the appropriate approach (33.3% always and 32.9% almost always) (Table 3). **Separate storage of raw meat or chicken from cooked food and food eaten raw** was stated by 41% of the women from Jordan (vegetables and salad above meat or chicken) [7] and 89% of consumers from Lebanon (raw chicken or meat separate from other cooked food) [46]. Italian students, parents and teachers most often put fruits and vegetables in the refrigerator drawer (69%), milk (62%), and eggs (49%) in the refrigerator door, cooked meat (46%), and cheese (38%) on the middle shelf and fish on the top shelf (32%) [12]. Issues with refrigerator storage that were observed in Italy included incorrect or random placement of food, placing similar items on different shelves, and overcrowding shelves or drawers [19]. However, the study emphasized that these challenges are not only due to consumers’ lack of knowledge and lack of uniform refrigerator temperature, but also due to refrigerator designs prioritizing esthetics over food safety [19].

##### Refrigerator Temperature

Storing food at the **appropriate temperature** is one of the key steps to maintain food safety. CFSS participants showed very good **knowledge** on the importance of storing food at appropriate temperature to ensure food safety as over 98% either strongly agreed or agreed with this (Table 2). Also in other countries, consumers know that food storage at appropriate temperatures is important for food safety. In Bangladesh, 75% of mothers of young children agreed that perishable foods should be refrigerated [49]. Knowledge of the connection between food storage at the appropriate temperature and prevention of foodborne disease was reported for consumers in Bangladesh (11%) [22] and Sweden (89% of university students) [29]. Knowledge of refrigerator food storage contributing to the delayed microbial growth in food was reported for consumers in the United Arab Emirates (43% of women [11] and 44% of women [41]), Poland (89%) [50], Thailand (93%) [50], and Lebanon (most) [51]. Most consumers in Ireland knew that during refrigeration pathogens present in the food can grow (34%), some thought pathogens do not grow (30%), and others believed that pathogens are killed by the cold temperature (14%) or did not know about this (22%) [4].

The vast majority (93%) of CFSS participants showed a positive **attitude** towards **storing food in accordance with the instructions** on the packaging, as this is either very important (45%) or important (47%) for them (Table 4). **Storing food at appropriate temperatures** is important for 88% of CFSS participants (33% very important and 55% important) (Table 4).

Refrigerator temperatures recommended by national and international public health organizations are below 4 °C or 5 °C. The Slovenian National Institute of Public Health (NIJZ) [52] and World Health Organization (WHO) [53] both recommend the refrigerator temperature to be below 5 °C. In the USA, the United States Department of Agriculture (USDA) [54] and Food and Drug Administration (FDA) [55], recommend the temperature to be 4.4 °C or below.

The knowledge of Slovenian consumers about recommended temperatures for food storage in the refrigerator should be improved. Over 27% of CFSS participants selected the 4 °C and over 19% selected 5 °C, but there were also over 20% that selected 8 °C (Table 5). Together, over 47% of CFSS participants selected temperatures higher than 5 °C. The high percentage of consumers (almost 41%) selecting higher temperatures (6–8 °C) could be connected with the storage instructions on some perishable foods stating the appropriate storage temperature range (e.g., 4–6 °C, 4–8 °C, 0–6 °C). The examples of these temperatures were observed on three foods (fruit yogurt, natural yogurt, and fresh milk) found on the Slovenian market. Considering the diversity of temperature ranges written as instructions on perishable food products, it is not surprising that consumers have different opinions about appropriate refrigerator temperatures and that these temperatures differ from the temperatures recommended by public health organizations. 

Good knowledge on correct refrigerator temperature was reported for consumers in Egypt (4%: 4 °C) [24], the United Arab Emirates (25% of women: 1–4 °C) [11], Bangladesh (27%: not higher than 4 °C [43] and 37%: 1–5 °C [26]), Malaysia (28%: 4 °C or lower) [56], Lebanon (30% [25], 49% [51], 77% [31]), Jordan (40% of women: 1–4 °C) [7], Italy (44%: 0–4 °C) [20], the United Arab Emirates (24% of women: 1–4 °C) [41], Slovenia (48% of elderly consumers: 1–4 °C) [13], the USA (51% of university students: 4.4 °C or below [9], 79% of parents of 2–3 years old children [47]), New Zealand (52%: 4–7 °C) [38], Sweden (59% of university students: 4–5 °C) [29], Serbia (68% of university students: 4 °C) [23], and Ireland (82%: 4 °C) [4], as well as 77% student dietitians (<5 °C: 97% in Lebanon, 88% in the UK, 63% in the USA) [44].

##### Checking Refrigerator Temperature and Expired/Spoiled Food

Most CFSS participants check their refrigerator temperature on the screen (almost 46%) and over 18% use a thermostat, but many participants (almost 27%) do not check the temperature (Table 1). Only 8% of the participants actually use a thermometer to measure the temperature in their refrigerator (Table 1). Thermometers were used by consumers to measure refrigerator temperature also in the USA [10], Slovenia (30%) [42], Egypt (32%) [24], and Serbia (54–55% of university students) [23]. Some other studies **only reported if the refrigerator temperature was checked and how often, but not the methods used**. Among Italian consumers, 58% stated that they check their refrigerator temperature [20]. The frequency of refrigerator temperature measuring ranged from **always** (only 11% of Slovenian elementary school children when opening the domestic refrigerator [15]), **regularly measuring** (9% of consumers from Lebanon [25]), **frequently measuring** (most consumers from China [28]), **daily measuring** (45% of consumers from Lebanon [31] and 41% of consumers from Lebanon: 22% daily, 11% two times per day, 8% three times per day [51]), and **never measuring** (59% consumers from Lebanon [51], 80% of Slovenian elderly [13], and most Slovenian women (79% nonpregnant, 79% pregnant, and 81% postpartum) [14]).

Weekly checking of the refrigerator for spoiled or expired food was the most common practice among almost 58% of CFSS participants (Table 1). Checking refrigerated foods was a daily practice for almost 27% and a monthly practice for over 8% of CFSS participants (Table 1). Two thirds of (66%) of Italian consumers stated they had cleaned their refrigerator in the last 3 weeks [20].

#### 3.2.4. Food Labeling

##### Food Labels

CFSS participants strongly agreed (34%) and agreed (52%) that food labels contain information important for ensuring food safety (Table 2). However, regarding the instructions for proper food handling being clear enough, only 9% strongly agreed and 44% agreed, but 32% neither agreed nor disagreed (Table 2). The importance of food label information (FLI) has also been observed for consumers in Bangladesh (56%: FLI is very important) [57], Poland (68%: storage instructions are relevant) [39], Slovenia (72% nonpregnant, 75% pregnant and 81% postpartum women: FLI has important instructions) [14], Bangladesh (87%: expiration dates are the most important part of FLI [57], and Saudi Arabia (99%) [58]. Consumers from the UK and Ireland [21], Bangladesh [57], Egypt [59], and Indian university students [60] also shared several aspects of labels that should be improved, including (1) legibility and readability issues [57,59,60], (2) information accessibility [21,57], (3) usefulness and comprehension [59], and (4) presentation and design preferences [59,60].

For the most part, CFSS participants followed the label information: 70% followed instructions for storage and preparation (22% always and 48% almost always), 80% followed the “Best before” expiration date (27% always and 53% almost always), 85% followed the “Use-by” expiration date (35% always and 50% almost always) (Table 3). A smaller number of CFSS participants indicated that they only sometimes followed the instructions (24%), “Best before” (15%), and “Use-by” (12%) (Table 3). Practices of reading food labels and food safety related information on the labels were reported for consumers in Bangladesh (53% [57] and 67% [43]), the United Arab Emirates (58% of women [11] and 74% of women [41]), Türkiye (87%) [61], Slovenia (54%) [40], Lao People’s Democratic Republic (64%) [34], Thailand (89%) [50], Poland (90%) [50], and the UK and Ireland (majority) [21]. The label information most frequently read included (1) storage instructions, (2) preparation instructions, and (3) expiration dates. Storage instructions were read by consumers in the United Arab Emirates (23% [11] and 35% of women [41]), India (44% of university students) [60], Bangladesh (51%) [43], Poland (67%) [39], Lebanon (83%) [46], the UK and Ireland (88%) [21], Türkiye (88%) [61], and China (most) [28]. Preparation instructions were read by consumers in the UK and Ireland (78%: cooking time) [21], Lebanon (83%) [46], Türkiye (86%) [61], and China (most) [28]. Expiration dates were checked by consumers in the United Arab Emirates (23% of women [11] and 35% of women [41]), Bangladesh (31% of mothers of young children) [49], the UK and Ireland (32%) [21], Slovenia (54%) [40], Türkiye (58%) [36], Brazil (64%) [45], Egypt (77%) [59], Thailand (89%) [50], Poland (90%) [50], Lebanon (96%) [46], and India (98% of university students) [60].

##### Dates of Durability (Expiration Dates)

The date of durability, more often colloquially called the expiration date, is the date until which properly stored food will keep its specific properties [62]. The date of durability is a part of the mandatory food label information in the European Union [62]. Dates of durability/expiration dates “Best before” and “Use-by” are connected to two different concepts. “Best before” indicates the date of minimum durability that the food retains the expected quality. “Use-by” indicates the date that the food is safe to use. CFSS participants showed very good knowledge about the meaning of these two terms: nearly 80% knew the different meanings of “Best before” and “Use-by”, but 19% believed that “Best before” is related to safety and “Use-by” is related to quality and less than 1% stated that they do not know the answer (Table 6). Most (70%) of student dietitians from the UK (81%), the USA (75%), and Lebanon (30%) were aware that the “Use-by” date is the best food safety indicator [44].

CFSS participants mostly have a positive attitude (31% very important and 51% important) towards following food expiration dates (Table 6).

The practices of CFSS participants handling expired food were diverse. For food with expired “Use-by” dates, 51% CFSS participants decided what to do based on smell, taste, and appearance, almost 30% discarded the food, and 12% used the food as soon as possible (Table 7). For food with expired “Best before” dates, 61% CFSS participants also decided based on smell, taste, and appearance, but over 25% used it as soon as possible and almost 10% discarded the food (Table 7). In New Zealand, only 48% of consumers used the “Best before” and “Use-by” dates to decide whether to consume foods stored in the refrigerator [38]. The majority of Chinese consumers usually used packaged food before its “Use-by” date expired [28]. Most consumers from the UK and Ireland were convinced they abide by the “Use-by” date, but their observed behavior revealed that the “Use-by” dates did not receive that much attention at the start of food preparation [21]. The practice of not using food after the expiration date was reported for consumers in the USA (35%—for bagged salad or fresh cut fruits showing no damage) [10], Brazil (60%) [45], Bangladesh (68% of mothers of young children) [49], Lebanon (79%) [46], Brazil (97%) [63], and Malaysia (most consumers) [8]. More than 80% of consumers in Türkiye never used milk and dairy products (94%), fish (94%), chicken meat (93%), red meat (88%), and eggs (84%) after expiration dates, but less than half of consumers never used vegetables (45%) and fruits (44%) after expiration dates [61]. Interestingly, 84% of Swedish students decided to use milk with an expired “Best before” date if it smelled and tasted well, but there was also 1% who used the milk regardless of date, smell, and taste [29].

#### 3.2.5. Food Preparation

Important ways of maintaining food safety during food preparation include the following: (1) washing fruits and vegetables before use, (2) not washing raw meat/poultry, (3) not preparing food with symptoms of illness, and (4) preventing cross-contamination during cutting of foods (appropriate uses of knives and chopping boards).

##### Washing Fruits and Vegetables

The majority of CFSS participants had good knowledge (80% strongly agreed and 17% agreed) about needing to **wash fresh fruits and vegetables** that are not peeled **before use** (Table 2). All (100%) mothers of young children (up to two years old) in Bangladesh thought that fruits and vegetables should be washed before eating [49]. Knowledge about fruits and vegetables needing to be washed under running water before use in order to prevent food poisoning was reported for consumers: Thailand (vegetables: 47%, fruits: 65%) [50], Bangladesh (53%) [22], Jordan (56% of women) [7], Poland (vegetables: 79% and fruits: 76% [50]; fruits and vegetables: 75% [39]), Saudi Arabia (89%) [58], the USA (97% university students) [9], and Romania (most consumers) [6].

Most CFSS participants reported that they always wash unpeeled fruits and vegetables before consumption: 81% when eaten raw and 75% when heat-treated (Table 3). Additionally, 15% (raw) and 16% (heat-treated) stated they almost always wash them. A smaller proportion reported washing them sometimes: 3% (raw) and 6% (heat-treated) (Table 3). Only a very small number of participants indicated that they mostly do not wash unpeeled fruits and vegetables (<1% raw and 2% heat-treated) or never wash them (<1% raw and heat-treated) (Table 3). One observational study of Slovenian elderly found that 69% chopped unwashed lettuce, 63% chopped unwashed cabbage (wiped with a multipurpose dishcloth), and only 6% chopped unwashed tomatoes [13]. A multimethod study from Italy reported the observation that washing fruits and vegetables was not performed in 1 (7%) of 14 families [19]. Washing of fruits and vegetables under running water before use was reported for consumers in Mexico (17% vegetables and 48% fruits) [64], Thailand (56%) [50], Brazil (57%) [45], the United Arab Emirates (61% women) [11], the Republic of Korea (67% in 2010 and 55% in 2019 always washed) [30], Egypt (69%) [24], Serbia (71% university students) [23], Poland (84% [39] and 93% [50]), and the USA (98% of the parents of elementary school children from Texas) [65].

Some studies also reported on the knowledge and practices of using other things besides water to wash fruits and vegetables, as well as unsafe practices of wiping or eating unwashed produce picked up from the ground on trips. In Lebanon, 19% of consumers knew that drinking water cannot be used to sterilize vegetables and fruits [46], but only 31% of consumers in Brazil knew that raw fruits and vegetables should not be washed using bleach before eating [45]. In Mexico, a number of consumers either combined washing and disinfecting (26% fruits and 34% vegetables) or only used disinfecting (22% fruits and 45% vegetables) to clean fruits and vegetables [64]. Among Serbian university students, 17% washed fruits and vegetables with soap, 6% washed them in salt and water, and 4% boiled fruits and vegetables [23].

##### (Not) Washing Raw Meat/Poultry

Washing raw poultry poses a significant risk due to the high prevalence of *Campylobacter* in poultry meat, as it can lead to aerosolized contamination of kitchen surfaces with bacteria. Public health institutions in different countries agree that raw poultry should not be washed, but their recommendations on not washing raw meat are not uniform. The Slovenian hygiene recommendations for food safety for consumers advise only not to wash raw poultry, but also comment that raw unprocessed foods of animal origin such as red meat, poultry, and fish can be a source of food poisoning when not handled properly [52]. Public health institutions from the USA [66,67], Scotland [68], and the UK [69] advise not to wash raw poultry and raw red meat.

CFSS participants varied in their belief that **washing raw meat and poultry** helps to ensure food safety: 32% strongly agree, 26% agree, 19% who neither agree nor disagree, and 16% disagree with this statement (9% disagree and 7% strongly disagree) (Table 2). Many consumers mistakenly believe that washing raw meat/poultry prevents foodborne illnesses. In Romania, most consumers believed that washing poultry is required to prevent foodborne disease [6], while in Ireland, consumers who washed chicken did so to be safe, removing the blood and the odor [4]. In eight Southeast Asian countries (Brunei, Cambodia, Indonesia, Lao People’s Democratic Republic, Malaysia, the Philippines, Thailand and Vietnam), only 2% of consumers knew that not washing raw poultry helps prevent cross-contamination in the kitchen [70] and over 71% were taught to wash poultry from family and friends [70]. Similarly, low awareness was reported in Malaysia, where only 3% of consumers knew that raw poultry should not be washed before preparation [8]. Knowledge was slightly better in Italy and the USA: 24% of Italian students and 30% of teachers thought chicken should not be washed [12], and 34% of university students from the USA knew that raw meat and poultry should not be washed [9]. However, most older adults from the USA who washed raw poultry were unaware that it was recommended not to do so [18].

CFSS participants had diverse attitudes towards washing raw red meat and poultry before heat treatment, as this was very important for 27% (red meat) and 34% (poultry), important for 26% (red meat) and 24% (poultry), neither important nor unimportant for 20% (red meat) and 16% (poultry), unimportant for 11% (red meat) and 10% (poultry), and not important at all for 9% (red meat) and 9% (poultry).

Among CFSS participants, the practice of washing raw meat and poultry was less frequently noted compared to the practice of washing fruits and vegetables. Meat was always washed by 33% and poultry by 40% of participants (Table 3). Additionally, 18% almost always washed meat and 16% almost always washed poultry (Table 3). Fewer participants sometimes washed meat (14%) and poultry (12%) (Table 3). Meat was mostly not washed by 14% and poultry by 11% (Table 3). Meat was never washed by 17% and poultry by 16% (Table 3). Washing raw meat or poultry was reported for consumers in the USA (33% of elderly—poultry [18]; parents of elementary school children from Texas: 86%—poultry [65], 75%—meat [65]), Southeast Asia (96%—poultry) [70], Slovenia (94% of elderly—chicken) [13], Ghana (98%—chicken) [17], Lao People’s Democratic Republic (58%—meat) [34], and Malaysia (most consumers—meat) [8]. In two cases, consumers did not only use water for washing, but also used salt for washing raw poultry (59% in Southeast Asia) [70] and in another case bleach, citrus juice, salt, or vinegar for washing meat (46% of parents of elementary school children from Texas, USA) [65]. In Ireland, 69% of consumers do not wash raw chicken [4], and in Lebanon, 53% of consumers do not wash raw meat [46].

##### (Not) Preparing Food with Symptoms of Illness

Food safety knowledge about consumers preparing food while exhibiting symptoms of illness varied across countries. The vast majority of CFSS participants knew that people with **symptoms of illness** can spread the infection **during food preparation** and pass it to those who consume the food (71% strongly agree and 23% agree) (Table 2). In the United Arab Emirates, 88% of women knew that diseases can be transmitted through food [11]. When asked “who should not prepare food for other people”, most Serbian students replied a person with a cold (46–51%), and only 10% replied a person with diarrhea [23]. Most consumers in China (73%—2019 and 2023) believed that a person with diarrhea should not prepare food for others, even if they washed their hands [28]. Similarly, 53% of consumers from Bangladesh knew that conditions such as diarrhea, fever, sore throat, or flu should prevent someone from cooking for others, but opinions on cooking with hand wounds were mixed: only 19% knew that person should wear gloves, while 21% thought the person can prepare food if the wound is not infected, 28% thought the person should not cook, and 33% thought a bandage on the wound would suffice [22]. Another study from Bangladesh found that less than half of consumers (42% pre-COVID-19 and 45% post-COVID-19) knew that uncovered abrasions or cuts can cause cross-contamination [26]. In contrast, most Chinese (89%—2019, 84%—2023) [28] consumers thought that hand injuries or cuts need to be covered to prevent or decrease the risk of food cross-contamination, and 75% of Slovenian elementary school children [15] and 89% of consumers in Lebanon thought that they should not prepare food with injured hands [25].

Cooking when having symptoms of illness is not a common practice for CFSS participants, as only 6% answered always and 6% almost always (Table 3). There were 14% who do so sometimes (Table 3). The majority of CFSS participants do not cook (31%) or never cook (41%) in such situations (Table 3). Other studies investigated the practices of cooking when having diarrhea, being sick, or having sores on hands. In Sweden, 9% of university students cooked for someone else when they had diarrhea [29]. In Lebanon, 80% of consumers do not prepare raw meat when sick [31]. Different actions were reported regarding cooking with a sore on the hand. Consumers in the following countries would continue to prepare food after treating the wound: Egypt (14%—wrap the wound in gauze and use a glove) [24], the Republic of Korea (75% in 2010 and 72% in 2019) [30], and Serbia (75–79% of university students: 34–35% a bandage, 28–29% both a bandage and a glove, 12–15% a glove) [23]. Consumers in Serbia (18–23% of university students) [23] and the Republic of Korea (6% in 2010 and 4% in 2019) [30] would not prepare food until the sore had healed. Consumers in Slovenia (26% of elementary school students) [15] and Bosnia and Herzegovina (74%) [27] would not cook with an opened/unprotected hand wound. In the Republic of Korea, consumers (20% in 2010 and 25% 2019) would continue cooking without treating the wound [30].

##### Preventing Cross-Contamination with Appropriate Uses of Knives and Chopping Boards

Appropriate use of knives and chopping boards during food preparation is crucial to prevent cross-contamination during the preparation of ingredients. The majority of CFSS participants have positive attitudes not to cut fresh vegetables with the same knife on the same chopping board previously used to cut raw meat, as 76% believed this was very important and 19% believed this was important (Table 4).

CFSS participants have good practice for the preparation of meat/poultry/fish and a salad with various vegetables as more than half selected the answer with the use of a separate knife and chopping board for meat and a separate knife and chopping board for vegetables (Table 1). There were also 24% of CFSS participants who use the same knife and chopping board but wash them with a dish detergent before cutting vegetables. A smaller number of CFSS participants use the same knife and chopping board and rinse them under hot (12%) or cold (4%) water before cutting vegetables. The concerning practice of using the same utensils without washing first for meat and then for vegetables was reported by 1.5% of CFSS participants. Previous studies described that a separate knife and a chopping board were used by consumers in Slovenia (10%: for cutting vegetables and then ready-to-eat food) [40], Sweden (39% of university students—for raw meat and salad vegetables) [29], Italy (47%) [20], and Türkiye (approximately 50% for raw and cooked fish [36] and 71% for meat and vegetables [61]). Studies also described that separate chopping boards were used by consumers in Mexico (24%) [64], Bangladesh (25% for raw meat, poultry, seafood and vegetables) [43], Lao People’s Democratic Republic (38% for raw and ready-to-eat foods and 43% for meat and vegetables) [34], Saudi Arabia (43% of women: for raw meat and cooked or ready-to-eat food) [16], Slovenia (55%: after handling raw meat [42], 66% for different foods [32] and most women—for raw and cooked foods, for raw meat and raw vegetables [14]), Serbia (51–61% of university students: for raw meat and tomatoes) [23], and Bosnia and Herzegovina (68% for raw meat and vegetables) [27]. It was reported that consumers used a separate knife in Mexico (28%) [64], the U.S. Virgin Islands (30%—raw meat and ready-to-eat foods) [37], and Saudi Arabia (36% of women: for raw meat and cooked or ready-to-eat food) [16]. Some consumers either used another knife/chopping board or washed them with detergent and hot water, which was found in Slovenia (78%—knife) [42], New Zealand (78%—chopping board and knife: for other food after preparing raw poultry) [38] and Malaysia (most consumers—chopping board) [8]. From a food safety perspective, using a different chopping board and knife for different foods is not the only safe approach. It is also acceptable to use the same chopping board and knife if they are first washed with detergent and hot water before further use. Washing these utensils with hot water and detergent was reported to be the practice of consumers in Sweden (37% of university students) [29], Slovenia (83%) [42], Ireland (94%—after cutting raw chicken) [4], and China (most consumers—after raw meat or chicken) [28]. After washing with detergent and water, the same chopping board was used by consumers in Slovenia (26%—for cutting vegetables and then ready-to-eat food [40], 44% of elderly consumers [13] and most women—after raw meat before heat-treated meat [14]), Mexico (39%) [64], Serbia (36–46% of university students—for raw meat and then for tomatoes) [23], Saudi Arabia (53% of women—cutting raw meat and cooked or ready-to-eat food) [16], and the USA (89% of parents of elementary school children from Texas—after meat before food consumed raw [65]). After washing with detergent and water, the same knife was used by consumers in the following countries: Slovenia (24%—for cutting vegetables and then ready-to-eat food [40] and 38% of elderly [13]), Jordan (35% of the women—after raw meat before vegetables) [7], Mexico (38%) [64], the U.S. Virgin Islands (56%—raw meat and ready-to-eat foods) [37], Saudi Arabia (60% of women—cutting raw meat and cooked or ready-to-eat food) [16], and Serbia (76–78% of university students—after cutting raw meat) [23].

There are many unsafe approaches to using chopping boards and knives when preparing food that can lead to cross-contamination. Examples of unsafe approaches mentioned in previous studies were diverse and included the following: using the opposite side of the chopping board, only washing or rinsing with water, and using the same knife and chopping board—perhaps wiping them with a towel. The use of the opposite side of the chopping board was stated by 5% of Swedish university students [29]. Washing and rinsing without detergent was performed by consumers in many countries: Ireland (knife and chopping board: 1% cold / 5% warm water, wiped dry) [4], Sweden (5% of university students: knife) [29], the U.S. Virgin Islands (12%—knife for raw meat and ready-to-eat foods) [37], Serbia (15–18% of university students—knife with cold water after cutting raw meat) [23], Mexico (21% knife and 20% chopping board) [64], and Italy (some families: chopping board with cold water) [19]. Some consumers keep using the same knife and/or chopping board (without cleaning): (1) both were reported in Mexico (13% the same knife and 17% the same chopping board) [64], Slovenia (48%—poultry preparation) [32], Türkiye (approximately 50% for raw and cooked fish) [36], and Italy (53% washed or unwashed) [20]; (2) the same chopping board in Bangladesh (27% always for raw meat, poultry, seafood and vegetables) [43]; and (3) the same knife in the U.S. Virgin Islands (2% raw meat and ready-to-eat foods) [37] and Poland (48% always/usually cut first raw and then cooked meat) [39]. A very small part of consumers stated that they use the same chopping board and/or knife as they were or only wiped in Sweden (<1% university students use the same knife and chopping board and <1% of university students wiped with a towel) [29], Ireland (<1% knife and chopping board—wiped with a wet towel) [4], Serbia (2–4% of university students—wipe chopping board with a paper towel; for raw meat and then for tomatoes and 2–3% of university students—wipe knife with a cloth; after cutting raw meat) [23], and the U.S. Virgin Islands (8% wipe knife with a damp cloth or paper towel; after raw meat and then ready-to-eat foods [37].

### 3.3. Structural Equation Modeling (SEM) and the Relationships Between Knowledge (K), Attitudes (A), and Practices (P)—KAP

#### 3.3.1. Structural Equation Modeling (SEM) Assumptions

Five structural equation models (SEMs) were developed to test the hypothesized relationships between knowledge (K), attitudes (A), and practices (P) variables, each corresponding to specific sections and topics of the CFSS questionnaire. Model 1, related to the section ”Food shopping, transportation, and refrigeration”, examined the topic of the placement of foods in the refrigerator by evaluating the direct influence of the knowledge variable K15 on the practice indicators P8 and P9 (hypothesis (H): H1_1, H1_2). Model 2, addressing the topic of refrigerator temperature within the same section, assessed the effects of knowledge variables K16 and K17 on the attitudinal factor A5 and the practice variable P12, testing both direct and indirect relationships (H2_1–H2_5). Model 3, derived from the section ”Food labeling and food preparation”, focused on food labels and expiration dates by investigating the associations between knowledge variable K22, attitudinal indicator A11, and practice indicators P69 and P70, considering both mediating and direct effects (H3_1–H3_3). Model 4, addressing the topic of washing of fruits and vegetables, assessed the direct effects of the knowledge variable K24 on practice indicators P71 and P72 (H4_1, H4_2). Finally, Model 5, related to the topic of washing of raw red meat and poultry, analyzed the complex interactions among knowledge variable K25, attitudinal factors A13 and A14, and practice indicators P73 and P74, incorporating both direct and mediated pathways (H5_1–H5_8).

The SEM study for Models 1–5 tested four key assumptions required for SEM: multivariate normality, multicollinearity, sample size adequacy, and positive definiteness.

CFSS tested four important assumptions for the SEM, including multivariate normality, multicollinearity, sample size, and positive definiteness. Multivariate normality was assessed by regression analysis, which revealed that 0 out of 1621 participants were outliers according to Mahalanobis distances [71]. The multicollinearity assumption was not violated [72], with variance inflation factors (VIFs) less than 10 (1.150–2.517) and tolerances greater than 0.01 (0.397–0.869) [73]. The linearity assumption was not assessed due to the use of a Likert scale. In addition, the assumptions of linearity and homoscedasticity were tested and found not to be violated. The variance values were checked and all variables had similar variance levels (0.202–4.671).

The sample size was also checked using an online calculator [74]. The analysis showed that a minimum sample size of 700 participants was required to achieve a desired statistical power of 0.8, taking into account two latent variables and two observed variables, with an expected effect size of 0.3 and a probability level of 0.05. In addition, a minimum sample size of 1621 participants was required for the model structure.

#### 3.3.2. Structural Equation Modeling (SEM) Models

**Figure 2:**
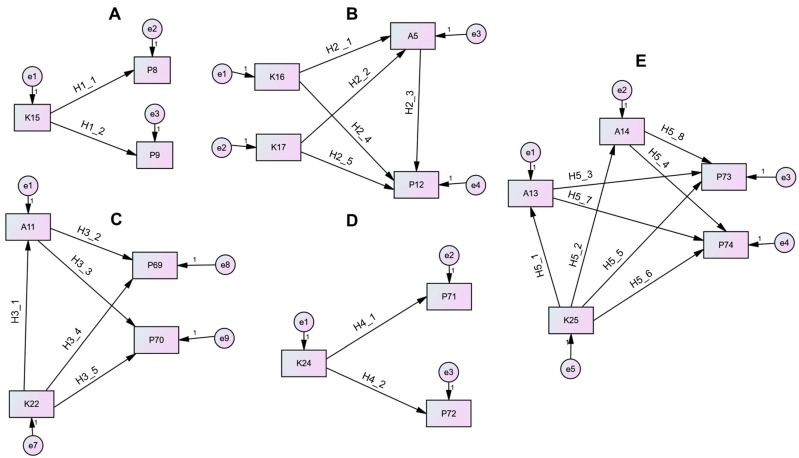
Structural equation models: Model 1 (**A**); Model 2 (**B**); Model 3 (**C**); Model 4 (**D**); Model 5 (**E**). All abbreviations for KAP questions are explained in Table 1, Table 2, Table 3, Table 4, Table 5, Table 6 and Table 7.

The SEM models were used to test how knowledge affects attitudes and practices (behavior), as well as how attitudes affect practices. These calculations allow for the examination of the overall fit of the model to the data and the simultaneous computation of all path coefficients [75,76]. The proposed models and hypotheses were evaluated using structural equation modeling (Figure 2). An overview of the models and food safety topics is presented in Table 8.

For **Model 1**, the findings from the SEM analysis (Table 9) demonstrated strong positive associations between the variables “knowledge on separate refrigerator storage of fresh meat, poultry and fish on the lowest shelf or separate from cooked food and food eaten raw” (K15) and both “practice of separate refrigerator storage of food consumed raw and food requiring heat-treatment” (P8), with an estimate of 0.371 (standard errors = 0.02, critical ratios = 18.898, *p* < 0.001) and “practice of separate refrigerator storage of fresh meat, poultry and fish on the lowest shelf or separate from cooked food and food eaten raw” (P9), with an estimate of 0.499 (standard errors = 0.02, critical ratios = 24.971, *p* < 0.001).

The SEM analysis of **Model 2** (Table 9) indicated significant positive relationships between the variables “knowledge on importance of appropriate temperature for food safety” (K16) and “attitude towards the refrigerator temperature” (A5), with an estimate of 0.476 (standard errors = 0.042, critical ratios = 11.463, *p* < 0.001), and “knowledge on the recommended temperature for refrigerated storage of food” (K17) and “attitude towards the refrigerator temperature” (A5), with an estimate of 0.031 (standard errors = 0.009, critical ratios = 3.557, *p* < 0.001). Additionally, “attitude towards the refrigerator temperature” (A5) had a significant positive relationship with “practice of checking the temperature in the refrigerator” (P12), with an estimate of 0.228 (standard errors = 0.029, critical ratios = 7.785, *p* < 0.001). In contrast, the relationships between the variables “knowledge on importance of appropriate temperature for food safety” (K16) and “practice of checking the temperature in the refrigerator” (P12), with an estimate of −0.035 (standard errors = 0.051, critical ratios = −0.69, *p* = 0.49), and “knowledge on the recommended temperature for refrigerated storage of food” (K17) and “practice of checking the temperature in the refrigerator” (P12), with an estimate of 0.013 (standard errors = 0.01, critical ratios = 1.236, *p* = 0.216), are not statistically significant. This indicates that while “knowledge on importance of appropriate temperature for food safety” (K16) and “knowledge on the recommended temperature for refrigerated storage of food” (K17) positively affect “attitude towards the refrigerator temperature” (A5), and “attitude towards the refrigerator temperature” (A5) positively affects “practice of checking the temperature in the refrigerator” (P12), there are no direct significant effects of “knowledge on importance of appropriate temperature for food safety” (K16) and “knowledge on the recommended temperature for refrigerated storage of food” (K17) on “practice of checking the temperature in the refrigerator” (P12).

According to the SEM analysis of **Model 3** (Table 9), “knowledge on food labels containing information important for ensuring food safety” (K22) significantly predicted “attitude towards following expiration date on food” (A11), with an estimate of 0.078 (standard errors = 0.023, critical ratios = 3.427, *p* < 0.001). The results also showed strong positive effects of “attitude towards following expiration date on food” (A11) on both “practice of following the date of expiration written as Best before” (P69), with an estimate of 0.726 (standard errors = 0.021, critical ratios = 34.614, *p* < 0.001), and “practice of following the date of expiration written as Use by” (P70), with an estimate of 0.646 (standard errors = 0.021, critical ratios = 30.754, *p* < 0.001). In contrast, the direct path from “knowledge on food labels containing information important for ensuring food safety” (K22) to both “practice of following the date of expiration written as Best before” (P69), with an estimate of 0.009 (standard errors = 0.019, critical ratios = 0.459, *p* = 0.646), and to “practice of following the date of expiration written as Use by” (P70), with an estimate of 0.053 (standard errors = 0.019, critical ratios = 2.718, *p* = 0.007) was not significant.

The results of the SEM analysis for **Model 4** (Table 9) show that “knowledge on washing of unpeeled fruits and vegetables before use” (K24) significantly predicted both “practice of washing of fruits and vegetables that are unpeeled and eaten raw” (P71), with an estimate of 0.496 (standard errors = 0.02, critical ratios = 24.269, *p* < 0.001) as well as “practice of washing of fruits and vegetables that are unpeeled and heat treated” (P72), with an estimate of 0.513 (standard errors = 0.031, critical ratios = 16.807, *p* < 0.001).

For **Model 5**, the SEM analysis (Table 9) revealed strong positive effects between “knowledge on washing raw meat and poultry effecting food safety” (K25) and both “attitude towards washing raw red meat before heat treatment” (A13), with an estimate of 0.756 (standard errors = 0.016, critical ratios = 46.284, *p* < 0.001) and “attitude towards washing raw poultry before heat treatment” (A14), with an estimate of 0.797 (standard errors = 0.016, critical ratios = 50.615, *p* < 0.001). “Knowledge on washing raw meat and poultry effecting food safety” (K25) also directly predicted both “practice of washing raw red meat before cooking” (P73), with an estimate of 0.162 (standard errors = 0.025, critical ratios = 6.376, *p* < 0.001), and “practice of washing poultry before cooking” (P74), with an estimate of 0.161 (standard errors = 0.024, critical ratios = 6.699, *p* < 0.001). Moreover, “attitude towards washing raw red meat before heat treatment” (A13) strongly predicted “practice of washing raw red meat before cooking” (P73), with an estimate of 0.758 (standard errors = 0.02, critical ratios = 38.707, *p* < 0.001). Similarly, “attitude towards washing raw poultry before heat treatment” (A14) was significantly and positively related to “practice of washing poultry before cooking” (P74), with an estimate of 0.802 (standard errors = 0.019, critical ratios = 41.885, *p* < 0.001). Interestingly, the connection between “attitude towards washing raw red meat before heat treatment” (A13) to “practice of washing poultry before cooking” (P74), with an estimate of 0.026 (standard errors = 0.018, critical ratios = 1.4, *p* = 0.161) was not significant. However, the path from “attitude towards washing raw poultry before heat treatment” (A14) to “practice of washing raw red meat before cooking” (P73), with an estimate of 0.061 (standard errors = 0.02, critical ratios = 3.021, *p* = 0.003) was statistically significant.

The SEM analysis of five models verified that there is a significant influence of knowledge on attitudes, and also that both knowledge and attitudes influence practices, but the strength of these effects varies depending on the context. In most cases, knowledge had strong and direct effects on attitudes (e.g., K16, K17 → A5; K25 → A13, A14), while attitudes consistently predicted corresponding practices (e.g., A11 → P69, P70; A13 → P73, A14 → P74). Direct paths from knowledge to practice were sometimes significant (e.g., K15 → P8, P9; K25 → P73, P74) and sometimes nonsignificant (e.g., K16, K17 → P12; K22 → P69), suggesting that attitudes influence the effects of knowledge into practice. Taken together, these findings support the KAP framework, highlighting the important role of attitudes in the transfer of knowledge into safe food handling practices.

#### 3.3.3. Relationships Between Knowledge (K), Attitudes (A), and Practices (P)—KAP

In CFSS, **knowledge** was proven to affect **attitudes** on the topics of refrigerator temperature (Model 2), food labels and expiration dates (Model 3), and washing of raw red meat and poultry (Model 5). Evidence from other studies confirms this positive pathway. Studies in Malaysia [33], Laos [34], Romania [6], and Sweden [5] all found that higher levels of knowledge were associated with more favorable food safety attitudes. This also indicates that improved knowledge can influence the attitudes shifts in a positive direction. However, the pathway is not universal, as one study found no significant association between knowledge and attitudes [8]. Overall, these findings imply that although knowledge can often provide the foundation for shaping attitudes, there are additional factors, such as effort, food safety experience, and learning about food safety [6], that can influence how strongly knowledge affects attitude. Strengthening knowledge transfer in ways that motivate consumers appears to be essential for improving positive food safety attitudes.

**Knowledge** was found to significantly affect food safety **practices** in CFSS on the topics of placement of foods in the refrigerator (Model 1), washing of fruits and vegetables (Model 4), and washing of raw red meat and poultry (Model 5). On the topics of refrigerator temperature (Model 2) and food labels and expiration dates (Model 3), the effects of knowledge on practices were not confirmed. This variability reflects broader trends in the literature. One study in Romania reported a significant positive effect of knowledge on practices, suggesting that, as knowledge becomes enhanced, practices improve [6], whereas in Malaysia, the relationship between knowledge and practices was negative and insignificant [8]. In Laos, knowledge was shown to affect perceived behavioral control—the only factor directly affecting practices [34]. In Sweden, attitudes were found to influence the impact that knowledge has on practices [5]. These results show that while knowledge can lead directly to safer practice, it can also have an indirect effect through other factors such as attitudes or perceived control. Despite these complicated relationships, it is critical that consumers have good food safety knowledge. Educational strategies should not only focus on enhancing consumer knowledge, but also consider the other factors influencing the transfer of knowledge into practice.

The influence of **attitudes** on **practices** was also confirmed in CFSS for most of the topics: refrigerator temperature (Model 2), food labels and expiration dates (Model 3), and in part for the washing of raw red meat and poultry (Model 5). In Model 5, two sets of questions (one referring to raw red meat and the other referring to poultry) were included. The results from Model 5 showed that attitudes towards washing raw red meat affect the practices of washing raw red meat and the same was observed for washing poultry. However, the attitudes towards washing raw red meat did not affect the practices of washing poultry, but the attitudes towards washing of poultry did affect the practices of washing raw red meat. This suggests that the influence of attitudes on practices was strongest when the attitudinal and behavioral measures are closely contextually related. Comparable findings on the attitudes significantly positively affecting practices were reported in Malaysia [8], Romania [6], and Sweden [5]. This correlation between attitudes and practices shows that with better attitudes practices are also expected to improve. Although one Malaysian study did not find a strong effect of attitudes on intention of safe food handling practices [33], the broader pattern indicates that attitudes consistently and positively influence practices. In addition, attitudes appear to serve as a bridge between knowledge and practice, which reinforces the idea that fostering positive food safety attitudes can effectively lead to improved practices (behavioral change). Nonetheless, practical barriers such as time, facilities, or convenience may reduce the extent to which positive attitudes are translated into actual food safety practices.

### 3.4. Limitations

This study used voluntary convenience (snowball) sampling, which may introduce self-selection bias. However, the high proportion of female participants likely reflects national gender patterns in food handling. All data were self-reported via questionnaire, a standard method in KAP research, as observational measurements were not feasible for this large sample (*n* = 1621). While demographic factors such as age and education may influence KAP, analysis of these potential confounding variables in the SEM was beyond the scope of the current study. The questionnaire was reviewed by experts, and their feedback on themes, topics, and questions was incorporated, although no formal panel interviews were conducted. Despite these limitations, the large sample and the comprehensive Matrix of Consumer Food Safety framework enabled a robust assessment of consumer KAP in Slovenia and contribute important evidence to an underinvestigated European context.

## 4. Conclusions

This Consumer Food Safety Study provides a comprehensive assessment of Slovenian consumer food safety knowledge, attitudes, and practices, covering topics such as food-related habits, shopping and transportation, refrigeration, labeling, and preparation. Overall, the findings indicate mostly good levels of food safety knowledge, positive attitudes, and sound practices among participants. Nevertheless, certain areas require improvement, including use of cooler bags/insulated bags for food transport, knowledge of appropriate refrigerator temperature, regular refrigerator temperature checks, and practices related to washing raw meat or poultry. Structural equation modeling confirmed that knowledge influences attitudes and, in most cases, both knowledge and attitudes significantly impact safe food handling practices. This emphasizes the importance of including all three KAP aspects in educational campaigns aimed at enhancing consumer food safety culture. This study contributes to the growing understanding of how these three elements interact to shape consumer food safety culture, offering a valuable foundation for designing more effective public health interventions both within Slovenia and in broader contexts. The study also successfully applied a newly developed Matrix of Consumer Food Safety for use in questionnaire design. Beyond its practical application, the Matrix proved valuable as a conceptual and analytical framework, enabling a deep and multidimensional synthesis of the interrelated factors that influence consumer food safety. The use of snowball sampling may have led to a less uniform participant structure that included a majority of women and individuals with university education. However, a pragmatic choice to use snowball sampling enabled the substantial sample size (*n* = 1621 of participants completed the whole questionnaire). Although limited to adult consumers in Slovenia, this study contributes valuable findings to the underinvestigated area of consumer food safety in Central Europe. Importantly, the CFSS findings align with broader European efforts to strengthen consumer food safety awareness and reduce foodborne illness through improved consumer practices. Integrating Slovenian data into cross-European initiatives can provide a more comprehensive understanding of consumer behavior across the region and inform the design of targeted interventions. This study highlights several priorities for action: (1) enhancing consumer food safety awareness in Slovenia with educational programs addressing identified gaps in knowledge, attitudes, and practices; (2) applying the Matrix of Consumer Food Safety to pinpoint specific areas of intervention, such as food storage and food handling practices; and (3) using these insights to guide policy makers, educators, and public health campaigns in developing evidence-based strategies. Interventions to improve food safety practices could focus on promoting the use of cooler or insulated bags for transport, increasing awareness of safe refrigerator temperatures and regular monitoring, and enhancing safe handling of raw meat and poultry and be communicated using old and new media as well as food labels. Future research can build from this study by employing the Matrix of Consumer Food Safety to conduct longitudinal and cross-regional comparisons of consumer food safety, allowing for benchmarking, monitoring the effectiveness of interventions, and supporting harmonized European initiatives to improve consumer food safety.

## Figures and Tables

**Table 1:** Food handling practice questions and replies.

Questionnaire Topic	Question Number	Question	Replies	Frequency (Percentage)
Habits	P1	How would you describe your habits in terms of growing and buying food?	I eat mostly home-grown food.	189 (11.7%)
			I eat home-grown and bought food.	1079 (66.6%)
			I mostly eat bought food.	335 (20.7%)
			Other	18 (1.1%)
	P2	How would you describe your eating habits?	I mostly eat home-cooked food.	1181 (72.9%)
			I eat food prepared at home and food prepared outside the home (e.g., in restaurants and canteens) in approximately equal share.	415 (25.6%)
			I mostly eat food prepared outside the home (e.g., in restaurants and canteens).	15 (0.9%)
			Other	10 (0.6%)
	P3	How would you describe your habits regarding food preparation at home?	I mostly use basic ingredients.	1312 (80.9%)
			I use approximately the same share of basic ingredients and purchased pre-prepared meals.	290 (17.9%)
			I mostly use purchased pre-prepared meals.	12 (0.7%)
			I do not prepare food at home.	3 (0.2%)
			Other	4 (0.2%)
Food shopping, transportation	P10	What do you use most often when buying unpackaged fruit and vegetables in the store?	Disposable gloves and plastic bags	485 (29.9%)
			Plastic bags	523 (32.3%)
			Disposable gloves and reusable bags	127 (7.8%)
			Reusable bags	169 (10.4%)
			I do not use any of the above—I put unpackaged fruit/vegetables in the shopping basket/cart	82 (5.1%)
			I do not know, it depends, sometimes one thing and other times another	194 (12.0%)
			Other	41 (2.5%)
	P11	Estimate how long it takes from the checkout to your home when you buy frozen or perishable foods (e.g., dairy products, fresh meat, poultry and fish):	Up to 30 min	1338 (82.5%)
			From 30 min to 1 h	213 (13.1%)
			From 1 h to 1.5 h	16 (1.0%)
			From 1.5 h to 2 h	8 (0.5%)
			More than 2 h	0 (0.0%)
			Other	46 (2.8%)
Refrigerator	P12	How do you check the temperature in your refrigerator?	With a thermometer	129 (8.0%)
			With a thermostat	297 (18.3%)
			On the screen	741 (45.7%)
			I do not check	434 (26.8%)
			Other	20 (1.2%)
	P13	How often do you check if the food in the refrigerator has spoiled (e.g., rot, mold) or if its use by date has expired?	Daily	435 (26.8%)
			Weekly	936 (57.7%)
			Monthly	140 (8.6%)
			Annually	7 (0.4%)
			Never	21 (1.3%)
			Other	82 (5.1%)
Food preparation	P78	For lunch, you prepare meat/ poultry/fish and a salad with various vegetables. Which statement best describes your preparation process?	You cut meat/poultry/fish on the chopping board, then use the same knife and chopping board to cut vegetables.	25 (1.5%)
			You cut meat/poultry/fish on the chopping board, then rinse the knife and chopping board under COLD running drinking water and use them to cut vegetables.	65 (4.0%)
			You cut meat/poultry/fish on the chopping board, then rinse the knife and chopping board under HOT running drinking water and use them to cut vegetables.	196 (12.1%)
			You cut meat/poultry/fish on the chopping board, then wash the knife and chopping board with dish detergent and use them to cut vegetables.	390 (24.1%)
			You cut meat/poultry/fish with a knife (for meat) on one chopping board, and vegetables with another knife (for vegetables) on another chopping board.	842 (51.9%)
			Other	103 (6.4%)

**Table 2:** Food safety knowledge questions and replies.

Questionnaire Topic	Question Number	Question	Replies					
		Indicate to What Extent You Agree with the Following Statements:	Strongly Agree	Agree	Neither Agree nor Disagree	**Disagree**	**Strongly Disagree**	**Do Not Know**
			Frequency (Percentage)					
Food shopping, transportation	K13	During shopping/transport, it is necessary to ensure that wet foods are separated from dry foods.	696 (42.9%)	685 (42.3%)	155 (9.6%)	33 (2.0%)	8 (0.5%)	44 (2.7%)
	K14	During shopping/transport, it is necessary to take care that raw meat/poultry/fish do not come into contact with other foods.	986 (60.8%)	493 (30.4%)	90 (5.6%)	17 (1.0%)	4 (0.2%)	31 (1.9%)
Refrigerator	K15	It is recommended to store fresh meat, poultry and fish on the lowest shelf in the refrigerator or to separate from cooked food and food consumed raw.	755 (46.6%)	560 (34.5%)	149 (9.2%)	35 (2.2%)	7 (0.4%)	115 (7.1%)
	K16	Storing food at the appropriate temperature is important for food safety.	1278 (78.8%)	330 (20.4%)	11 (0.7%)	1 (0.1%)	0(0.0%)	1 (0.1%)
Food labeling	K22	Food labels contain information important for ensuring food safety (e.g., date of durability/expiration date, instructions for storage and food preparation).	557 (34.4%)	840 (51.8%)	171 (10.5%)	39 (2.4%)	7 (0.4%)	7 (0.4%)
	K23	The instructions for proper food handling on the food packaging are clear enough for you to handle the food properly.	153 (9.4%)	716 (44.2%)	522 (32.2%)	194 (12.0%)	26 (1.6%)	10 (0.6%)
Food preparation	K24	Fresh fruits and vegetables that are not peeled (e.g., apricots, peppers) must be washed before use.	1295 (79.9%)	271 (16.7%)	41 (2.5%)	7 (0.4%)	1 (0.1%)	6 (0.4%)
	K25	By washing raw meat and poultry we ensure food safety.	510 (31.5%)	426 (26.3%)	306 (18.9%)	149 (9.2%)	106 (6.5%)	124 (7.6%)
	K26	A person with symptoms of illness (e.g., vomiting, diarrhea, purulent wounds, discharge from the eyes/ears) can spread the infection during food preparation and pass it on to those who consume it.	1150 (70.9%)	379 (23.4%)	52 (3.2%)	9 (0.6%)	3 (0.2%)	28 (1.7%)

**Table 3:** Food handling practice questions and replies about frequency of different practices.

Questionnaire Topic	Question Number	Question	Replies					
		Mark How Often…	Always	Almost Always	Sometimes	Mostly Not	Never	Do Not Know
			Frequency (Percentage)					
Food shopping, transportation	P4	During shopping/transport, you place food so that wet food is separated from dry food.	496 (30.6%)	600 (37.0%)	243 (15.0%)	175 (10.8%)	91 (5.6%)	16 (1.0%)
	P5	During shopping/transport, you place food so that raw meat/poultry/fish are not in contact with other foods.	643 (39.7%)	524 (32.3%)	220 (13.6%)	133 (8.2%)	64 (3.9%)	37 (2.3%)
	P6	When shopping for frozen products and perishable foods (e.g., dairy products, fresh meat, poultry and fish), you add these products to the shopping basket/cart last (right before going to the checkout).	441 (27.2%)	538 (33.2%)	309 (19.1%)	223 (13.8%)	95 (5.9%)	15 (0.9%)
	P7	You use cooler bags/insulated bags to transport perishable foods (e.g., dairy products, frozen products, fresh meat, poultry and fish).	246 (15.2%)	346 (21.3%)	407 (25.1%)	314 (19.4%)	302 (18.6%)	6 (0.4%)
Refrigeration	P8	You place the food in the refrigerator in such a way that the food that is consumed raw is separated from the food that needs to be heat-treated (e.g., meat, fish, etc.).	572 (35.3%)	573 (35.3%)	257 (15.9%)	132 (8.1%)	68 (4.2%)	19 (1.2%)
	P9	You store fresh meat, poultry and fish on the lowest shelf in the refrigerator or separate from cooked food and food eaten raw.	539 (33.3%)	533 (32.9%)	279 (17.2%)	155 (9.6%)	74 (4.6%)	41 (2.5%)
Food labeling	P68	Read and follow the instructions for storage and use/preparation of food that are specified on the product label.	360 (22.2%)	770 (47.5%)	393 (24.2%)	74 (4.6%)	21 (1.3%)	3 (0.2%)
	P69	Follow the date of durability/expiration date written as “Best before”.	431 (26.6%)	860 (53.1%)	248 (15.3%)	71 (4.4%)	9 (0.6%)	2 (0.1%)
	P70	Follow the date of durability/expiration date written as “Use by”.	567 (35.0%)	811 (50.0%)	193 (11.9%)	40 (2.5%)	7 (0.4%)	3 (0.2%)
Food preparation	P71	Wash fruits and vegetables that you do not peel, if you eat them raw.	1316 (81.2%)	245 (15.1%)	44 (2.7%)	12 (0.7%)	2 (0.1%)	2 (0.1%)
	P72	Wash fruits and vegetables that you do not peel, if they are heat-treated.	1209 (74.6%)	271 (16.7%)	98 (6.0%)	26 (1.6%)	10 (0.6%)	7 (0.4%)
	P73	Wash raw red meat before cooking.	527 (32.5%)	286 (17.6%)	220 (13.6%)	227 (14.0%)	269 (16.6%)	92 (5.7%)
	P74	Wash the poultry before cooking.	651 (40.2%)	263 (16.2%)	187 (11.5%)	181 (11.2%)	264 (16.3%)	75 (4.6%)
	P75	You cook at home for members of your household even when you have symptoms such as vomiting, diarrhea, purulent wounds and discharge from the eyes/ears.	91 (5.6%)	101 (6.2%)	231 (14.3%)	506 (31.2%)	657 (40.5%)	35 (2.2%)

**Table 4:** Food safety attitude questions and replies about importance.

Questionnaire Topic	Question Number	Question	Replies					
		Indicate How Important It Is for You…	Very Important	Important	Neither Important nor Unimportant	Unimportant	Not Important at All	Do Not Know
			Frequency (Percentage)					
Food shopping, transportation	A2	To add frozen products and perishable foods (e.g., dairy products, fresh meat, poultry and fish) to the shopping basket/cart last (right before going to the checkout).	456 (28.1%)	612 (37.8%)	324 (20.0%)	167 (10.3%)	51 (3.1%)	11 (0.7%)
	A3	To take frozen products and perishable foods (e.g., dairy products, fresh meat, poultry and fish) home quickly after purchase.	1079 (66.6%)	506 (31.2%)	27 (1.7%)	4 (0.2%)	2 (0.1%)	3 (0.2%)
Refrigeration	A4	That you store food in accordance with the instructions on the packaging.	739 (45.6%)	767 (47.3%)	97 (6.0%)	14 (0.9%)	1 (0.1%)	3 (0.2%)
	A5	At what temperature is the food stored in your refrigerator.	534 (32.9%)	887 (54.7%)	160 (9.9%)	22 (1.4%)	4 (0.2%)	14 (0.9%)
Food labeling	A11	To follow the expiration date of the food.	601 (37.1%)	831 (51.3%)	149 (9.2%)	33 (2.0%)	6 (0.4%)	1 (0.1%)
Food preparation	A12	That you do not cut fresh vegetables (for salad) on the same board and with the same knife that you previously used to cut raw meat/poultry (without washing the board and knife).	1238 (76.4%)	300 (18.5%)	56 (3.5%)	11 (0.7%)	5 (0.3%)	11 (0.7%)
	A13	To wash raw red meat before heat treatment.	434 (26.8%)	421 (26.0%)	324 (20.0%)	176 (10.9%)	147 (9.1%)	119 (7.3%)
	A14	To wash poultry before heat treatment.	548 (33.8%)	395 (24.4%)	264 (16.3%)	160 (9.9%)	147 (9.1%)	107 (6.6%)

**Table 5:** Other food safety knowledge question and replies about recommended refrigerator temperature.

Questionnaire Topic	Question Number	Question	Replies	Frequency (Percentage)
Refrigeration	K17	Up to what temperature is it recommended to store food in the refrigerator (at home)?	0 °C	5 (0.3%)
			1 °C	7 (0.4%)
			2 °C	24 (1.5%)
			3 °C	63 (3.9%)
			4 °C	446 (27.5%)
			5 °C	311 (19.2%)
			6 °C	194 (12.0%)
			7 °C	137 (8.5%)
			8 °C	331 (20.4%)
			9 °C	12 (0.7%)
			10 °C	56 (3.5%)
			11 °C	7 (0.4%)
			12 °C	12 (0.7%)
			13 °C	4 (0.2%)
			14 °C	2 (0.1%)
			15 °C	10 (0.6%)

**Table 6:** Other food safety knowledge questions and replies about the meaning of “Use by” and “Best before”.

Questionnaire Topic	Question Number	Question	Replies		
		What Do You Think the Following Date of Durability/Expiration Date Labels Mean: “Use by” and “Best Before”?	The Date of Minimum Durability Until Which the Food Retains the Expected Quality	The Date Until Which the Food is Safe to Use	Do Not Know
			Frequency (Percentage)		
Food labeling	K27	Use by	317 (19.6%)	1294 (79.8%)	10 (0.6%)
	K28	Best before	1287 (79.4%)	323 (19.9%)	11 (0.7%)

**Table 7:** Food handling practice questions and replies about handling food with expired expiration dates.

Questionnaire Topic	Question Number	Question	Replies					
			I Throw It Away	I Use It as Soon as Possible	I Decide on the Use Based on Smell, Taste and Appearance	I Use It to Feed Animals	I Do Not Know	Other
			Frequency (Percentage)					
Food labeling	P76	What do you most often do with food with an expired “Use by” date?	481 (29.7%)	195 (12.0%)	823 (50.8%)	71 (4.4%)	5 (0.3%)	46 (2.8%)
	P77	What do you most often do with food with an expired “Best before” date?	158 (9.7%)	413 (25.5%)	988 (61.0%)	32 (2.0%)	5 (0.3%)	25 (1.5%)

**Table 8:** Overview of SEM models by food safety topics.

Model Number	Questionnaire Section	Food Safety Topic
1	3. Food shopping, transportation and refrigeration	Placement of foods in the refrigerator
2		Refrigerator temperature
3	5. Food labeling and food preparation	Food labels and expiration dates
4		Washing of fruits and vegetables
5		Washing of raw meat and poultry

**Table 9:** Parameters of the KAP SEM models 1–5.

Model	Hypothesis	Path	Estimate	S.E.	C.R.	*p*	Result
1	H1_1	P8<---K15	0.371	0.020	18.898	***	Confirmed
	H1_2	P9<---K15	0.499	0.020	24.971	***	Confirmed
2	H2_1	A5<---K16	0.476	0.042	11.463	***	Confirmed
	H2_2	A5<---K17	0.031	0.009	3.557	***	Confirmed
	H2_3	P12<---A5	0.228	0.029	7.785	***	Confirmed
	H2_4	P12<---K16	−0.035	0.051	−0.690	0.490	Not confirmed
	H2_5	P12<---K17	0.013	0.010	1.236	0.216	Not confirmed
3	H3_1	A11<---K22	0.078	0.023	3.427	***	Confirmed
	H3_2	P69<---A11	0.726	0.021	34.614	***	Confirmed
	H3_3	P70<---A11	0.646	0.021	30.754	***	Confirmed
	H3_4	P69<---K22	0.009	0.019	0.459	0.646	Not confirmed
	H3_5	P70<---K22	0.053	0.019	2.718	0.007	Not confirmed
4	H4_1	P71<---K24	0.496	0.020	24.269	***	Confirmed
	H4_2	P72<---K24	0.513	0.031	16.807	***	Confirmed
5	H5_1	A13<---K25	0.756	0.016	46.284	***	Confirmed
	H5_2	A14<---K25	0.797	0.016	50.615	***	Confirmed
	H5_3	P73<---A13	0.758	0.020	38.707	***	Confirmed
	H5_4	P74<---A14	0.802	0.019	41.885	***	Confirmed
	H5_5	P73<---K25	0.162	0.025	6.376	***	Confirmed
	H5_6	P74<---K25	0.161	0.024	6.699	***	Confirmed
	H5_7	P74<---A13	0.026	0.018	1.400	0.161	Not confirmed
	H5_8	P73<---A14	0.061	0.020	3.021	0.003	Confirmed

S.E.—standard errors; C.R.—critical ratios; *p*—probability value; *** *p* < 0.001. All abbreviations for KAP questions listed in column Path of this table are explained in Table 1, Table 2, Table 3, Table 4, Table 5, Table 6 and Table 7.

## Data Availability

The original contributions presented in this study are included in the article; further inquiries can be directed to the corresponding authors.

## Abbreviations

The following abbreviations are used in this manuscript:

A	Attitude
CFI	Comparative Fit Index
CFSS	Consumer Food Safety Study
C.R.	Critical Ratios
EFSA	European Food Safety Authority
FDA	Food and Drug Administration
FLI	Food Label Information
GFI	Goodness of Fit
H	Hypothesis
K	Knowledge
KAP	Knowledge Attitude Practice
MCFS	Matrix of Consumer Food Safety
NFI	Normed Fit Index
NIJZ	Slovenian National Institute of Public Health
P	Practice
*p*	Probability value
RMSEA	Root Mean Square Error of Approximation
S.E.	Standard Errors
SEM	Structural Equation Modeling
USDA	United States Department of Agriculture
WHO	World Health Organization

## Appendix A

**Table A1:** Matrix of Consumer Food Safety (MCFS) covering the questions presented in this paper.

Topic	Questions		
	Knowledge (K)	Attitude (A)	Practice (P)
**Habits**			**P1** How would you describe your habits in terms of growing and buying food?
			**P2** How would you describe your eating habits?
			**P3** How would you describe your habits regarding food preparation at home?
**Food shopping and transportation**	**K13** To what extent do you agree that during shopping/transport, it is necessary to ensure that wet foods are separated from dry foods?		**P4** How often during shopping/transport, do you place food so that wet food is separated from dry food?
	**K14** To what extent do you agree that during shopping/transport, it is necessary to take care that raw meat/poultry/fish do not come into contact with other foods?		**P5** How often during shopping/ transport, do you place food so that raw meat/poultry/fish are not in contact with other foods?
		**A2** How important it is for you to add frozen products and perishable foods (e.g., dairy products, fresh meat, poultry and fish) to the shopping basket/cart last (right before going to the checkout)?	**P6** How often when shopping for frozen products and perishable foods (e.g., dairy products, fresh meat, poultry and fish), you add these products to the shopping basket/cart last (right before going to the checkout)?
			**P7** How often do you use cooler bags/insulated bags to transport perishable foods (e.g., dairy products, frozen products, fresh meat, poultry and fish)?
**Refrigeration**	**K15** To what extent do you agree that it is recommended to store fresh meat, poultry and fish on the lowest shelf in the refrigerator or to separate from cooked food and food consumed raw?		**P8** How often do you place the food in the refrigerator in such a way that the food that is consumed raw is separated from the food that needs to be heat-treated (e.g., meat, fish, etc.)?
			**P9** How often do you store fresh meat, poultry and fish on the lowest shelf in the refrigerator or separate from cooked food and food eaten raw?
			**P10** What do you use most often when buying unpackaged fruit and vegetables in the store?
		**A3** How important is it for you to take frozen products and perishable foods (e.g., dairy products, fresh meat, poultry and fish) home quickly after purchase?	**P11** How long does it take from the checkout to your home when you buy frozen or perishable foods (e.g., dairy products, fresh meat, poultry and fish)?
		**A4** How important is it for you that you store food in accordance with the instructions on the packaging?	
	**K16** To what extent do you agree that storing food at the appropriate temperature is important for food safety?	**A5** How important is it for you at what temperature is the food stored in your refrigerator?	**P12** How do you check the temperature in your refrigerator?
	**K17** Up to what temperature is it recommended to store food in the refrigerator (at home)?		
			**P13** How often do you check if the food in the refrigerator has spoiled (e.g., rot, mold) or if its Use by date has expired?
**Food labeling**	**K22** To what extent do you agree that food labels contain information important for ensuring food safety (e.g., expiration date, instructions for storage and food preparation)?	**A11** How important is it for you to follow the expiration date of the food?	**P69** How often do you follow the date of expiration written as “Best before”?
			**P70** How often do you Follow the date of expiration written as “Use by”?
	**K23** To what extent do you agree that the instructions for proper food handling on the food packaging are clear enough for you to handle the food properly?		**P68** How often do you read and follow the instructions for storage and use/preparation of food that are specified on the product label?
**Food labeling**	**K27** What do you think the expiration date label “Use by” means?		**P76** What do you most often do with food with an expired “Use by” date?
	**K28** What do you think the expiration date label “Best before” means?		**P77** What do you most often do with food with an expired “Best before” date?
**Food preparation**	**K24** To what extent do you agree that fresh fruits and vegetables that are not peeled (e.g., apricots, peppers) must be washed before use?		**P71** How often do you wash fruits and vegetables that you do not peel, if you eat them raw?
			**P72** How often do you wash fruits and vegetables that you do not peel, if they are heat-treated?
	**K25** To what extent do you agree that by washing raw meat and poultry we ensure food safety?	**A13** How important is it for you to wash raw red meat before heat treatment?	**P73** How often do you wash raw red meat before cooking?
		**A14** How important is it for you to wash poultry before heat treatment?	**P74** How often do you wash the poultry before cooking?
	**K26** To what extent you agree that a person with symptoms of illness (e.g., vomiting, diarrhea, purulent wounds, discharge from the eyes/ears) can spread the infection during food preparation and pass it on to those who consume it?		**P75** How often do you cook at home for members of your household even when you have symptoms such as vomiting, diarrhea, purulent wounds and discharge from the eyes/ears?
		**A12** How important is it for you that you do not cut fresh vegetables (for salad) on the same board and with the same knife that you previously used to cut raw meat/poultry (without washing the board and knife)?	**P78** For lunch, you prepare meat/poultry/fish and a salad with various vegetables. What best describes your preparation process? [e.g., one (unwashed or washed with hot/cold water/detergent) or two knives and chopping boards]
